# Gender Differences in the Relationship Between Social Support and Quality of Life Among People Living with HIV During the COVID-19 Pandemic

**DOI:** 10.1089/whr.2024.0112

**Published:** 2024-11-25

**Authors:** Nikki Bhatia, Liying Wang, Francis Slaughter, Anh Tuyet Nguyen, Sarah Smith, Jane M. Simoni, Heidi M. Crane, Susan M. Graham

**Affiliations:** ^1^ Department of Anthropology, University of Washington, Seattle, Washington, USA.; ^2^ Department of Sociology, University of Washington, Seattle, Washington, USA.; ^3^ College of Nursing, Florida State University, Tallahassee, Florida, USA.; ^4^ Department of Epidemiology, University of Washington, Seattle, Washington, USA.; ^5^ Department of Medicine, Santa Clara Valley Medical Center, San Jose, California, USA.; ^6^ Center on Gender Equity and Health, University of California, San Diego, California, USA.; ^7^ Department of Psychology, University of Washington, Seattle, Washington, USA.; ^8^ Department of Gender, Women and Sexuality Studies, University of Washington, Seattle, Washington, USA.; ^9^ Department of Global Health, University of Washington, Seattle, Washington, USA.; ^10^ Department of Medicine, University of Washington, Seattle, Washington, USA.; ^11^ Department of Health Systems and Population Health, University of Washington, Seattle, Washington, USA.

**Keywords:** HIV/AIDS, mental health, social support, gender, health disparities, quality of life

## Abstract

**Background:**

The COVID-19 pandemic and related social distancing greatly impacted quality of life, in part, by disrupting access to social support. We examined the relationship between social support and quality of life among people living with HIV (PWH) who participated in a cross-sectional survey during the COVID-19 pandemic, evaluating differences in this relationship by age and gender.

**Materials and Methods:**

Between August 2020 and March 2021, 397 PWH completed an online Research Electronic Data Capture survey. Social support was assessed using the Multidimensional Scale of Perceived Social Support, and health-related quality of life (HRQoL) was assessed using the EuroQol EQ-5D-3L. Linear regression was used to examine the relationship between social support and quality of life, and interaction terms were used to assess effect modification by age and gender.

**Results:**

Higher levels of social support were associated with higher self-rated quality of life (adjusted β = 1.05, 95% confidence interval [95% CI]: 0.002, 2.10). This association was moderated by gender, with males having higher HRQoL (adjusted β = 24.5, 95% CI 10.5, 38.6) and a lower slope for the relationship between social support and HRQoL (adjusted β = −0.28, 95% CI −0.50, −0.06) than females. Age did not moderate the relationship between social support and quality of life, but higher age was associated with lower quality of life (adjusted β = −0.18, 95% CI −0.31, −0.05).

**Conclusion:**

Higher levels of social support were associated with better quality of life for PWH during the COVID-19 pandemic, especially for women. Our findings suggest that women may be more sensitive to large-scale interruptions in their social support networks than men.

## Background

The introduction of the SARS-CoV-2 virus into the United States in December 2019 and the ensuing rapid spread, morbidity, and mortality from COVID-19 drastically impacted all aspects of life, including social connections. In March of 2020, the Centers for Disease Control and Prevention (CDC) recommended social distancing (*i.e.,* staying at least 6 feet away from others and reducing close contact) to minimize SARS-CoV-2 transmission.^1^ Strict social distancing regulations introduced in Washington State and elsewhere were associated with mental distress. For example, a CDC study conducted in the United States in June 2020 found that younger adults and racial and ethnic minorities reported high levels of adverse mental health conditions, substance use, and suicidal ideation.^2^ An online survey in 2020 completed by a diverse population of 2020 individuals from around the world with a wide range of age, gender, income, education level, and occupation found that 60% of participants who experienced self-isolation, defined as staying away from other people to prevent the spread of the virus, reported a deterioration of their mental health after lockdown measures were enforced.^3^ Notably, those who were self-isolating at the time of responding to the survey scored higher on the Patient Health Questionnaire-9 (PHQ-9), indicating more severe depressive symptoms, than those who were not self-isolating.^3^

Social support is an important buffer between stressors and deteriorating mental health and can come from family, friends, and significant others. The American Psychological Association defines social support as “the provision of assistance or comfort to others, typically to help them cope with biological, psychological, and social stressors.”^4^ There are many different types of social support, including emotional support (*i.e.,* outward expressions of empathy and love), instrumental support (*i.e.,* tangible aid and service), informational support (*i.e.,* advice and suggestions), and appraisal support (*i.e.,* evaluative comments that could lead to self-improvement).^5^ All types of social support were potentially impacted by the isolation brought forward by the COVID-19 pandemic.^3^

Prior literature indicates that adequate social support is important for people living with HIV (PWH) and positively related to quality of life using a range of quality-of-life assessments, including those focused on behaviors, health, and subjective well-being.^6^ Since PWH are at an increased risk for depression and anxiety,^7^ adequate social support is even more important for this population’s health and well-being. Indeed, PWH with mental health disorders have been found to have lower quality of life than those without these conditions.^8^ PWH faced many challenges during the pandemic, with impacts on access to care, employment, income, housing, food insecurity, and increased stress, which many faced in situations of relative social isolation and loneliness.^9^ In a quantitative study of PWH conducted in Western Washington during the COVID-19 pandemic, we found that COVID-19-related stress was associated with higher depression and anxiety symptoms, but that greater social support was associated with lower depression symptoms.^10^ In a qualitative study based on interviews with a subset of participants in the quantitative study, we found that accessing social support was an important way that PWH coped with pandemic-related stressors.^11^ Prior research has shown that women living with HIV report lower quality of life than men living with HIV.^12,13^ In addition, as the number of PWH age 50 and older has increased, quality of life among older PWH has become an important focus of research and interventions.^14^

The goal of this study was to examine the relationship between social support and health-related quality of life (HRQoL) (*i.e.,* that related to the impact of health on a person’s ability to live a fulfilling life) during the COVID-19 pandemic among PWH in Western Washington. In addition, we aimed to determine whether age and gender moderated the relationship between social support and quality of life.

## Methods

### Study design and population

Participants were recruited from the University of Washington (UW) HIV patient registry, which supports the screening and recruitment of patients at UW-affiliated HIV care clinics in Western Washington into clinical research studies *via* a dedicated research nurse. Eligible individuals were 18 years of age or older, English-speaking, had internet access, and had consented to be contacted about research study participation. Survey recruitment was conducted through email, phone calls, and text messaging, depending on participant preference recorded in the registry. All participants provided electronic informed consent for study participation. Participants who completed the survey received a $20 electronic gift card.

### Data collection

Study data were collected and managed using REDCap (Research Electronic Data Capture), a secure, web-based software platform hosted at the UW.^15^ The REDCap survey collected data on sociodemographic characteristics, COVID-19 impact (pandemic stress, social stress, job and housing challenges), mental health symptoms, substance use, technology access, and the use of technological adaptations such as telemedicine. Additional questions assessed social support and quality of life, as detailed below.

### Measures

The following measures were used in this analysis.

- Sociodemographic characteristics: Age was calculated from date of birth (abstracted from the UW HIV patient registry) and the date of survey administration and was analyzed as a continuous variable to evaluate the hypothesis of a gradual decrease in quality of life with aging. Gender was assessed by asking participants which of the following genders they identified most closely with: female, male, transgender woman, transgender man, genderqueer/gender non-conforming, or other, with a prompt to specify their identity. For the purposes of this study, gender was analyzed in three categories as follows: female (including transgender females), male (including transgender males), and non-binary individuals (including genderqueer, gender non-conforming, and other identities). Biologic sex was defined as sex assigned at birth (male or female).
- Social support: Social support was assessed using the 12-item Multidimensional Scale of Perceived Social Support, a validated tool used to assess an individual’s perception of support from the following three sources: family, friends, and a significant other.^16^ Each question has response options on a 7-point scale ranging from “very strongly disagree” (1) to “very strongly agree” (7). The overall score for social support was calculated by summing the scores for each item and dividing by 12, for a possible range of 1–12, with a higher score representing a higher level of social support; subscores for each domain ranging from 1 to 7 were also calculated.^16^
- Quality of life: HRQoL was assessed using the EuroQol EQ-5D-3L questionnaire, which asks participants to rate their current state of health in terms of mobility, self-care, usual activity, pain, and anxiety or depression.^17^ Responses to the five items measured were scored on a scale of 1–3, with a lower total score indicating a higher HRQoL. An overall quality of life score was generated by summing the scores for each item. Scores were then weighted according to the EQ-5D US-based scoring and converted to a percentile.^18^
- Mental health: Depressive symptoms over the past 2 weeks were assessed using the PHQ-8 survey, which consists of eight questions with response options assessing symptom frequency, including “not at all,” “several days,” “more than half the days,” or “nearly every day,” and omits the PHQ-9 item on suicide and thoughts of self-harm.^19^ Anxiety symptoms over the past 2 weeks were assessed using the Generalized Anxiety Disorder-7 (GAD-7), which consists of seven questions with the same response options as for the PHQ-8.^20^ For both surveys, higher scores represented increasing symptom severity. Moderate-to-severe depression was defined as a PHQ-9 score ≥10, and moderate-to-severe anxiety was defined as a GAD-7 score ≥10.
- Household environment: Change in household crowding was measured by the following question: “Is your current living situation more crowded than before?” Response options were “yes” or “no.”

### Data analysis

Descriptive statistics were used to summarize characteristics of the study sample. Scatterplots were initially used to explore the relationship between variables, and pairwise correlation coefficients were calculated. Linear regression was used to evaluate the relationship between social support and HRQoL, before and after adjustment for potential confounders. Age and gender were considered *a priori* confounders. Potential additional confounders of the relationship between social support and HRQoL that were evaluated included race/ethnicity, depressive symptoms, anxiety, and a more crowded living situation than before the pandemic. Multivariable models included age and gender *a priori*, as well as other factors associated with the outcome at *p* < 0.20 in bivariable analysis. Interaction terms between social support and age and between social support and gender were also tested, to determine whether age or gender moderated the relationship between social support and HRQoL. Margin plots were created using Stata’s “marginsplot” command and used to examine results graphically. A sensitivity analysis using sex at birth rather than gender was also performed.

### Ethical review

The study protocol and instruments were reviewed and approved by the UW Human Subjects Division (STUDY00010385). All participants provided electronic informed consent.

## Results

Table 1 presents demographic characteristics of the 397 participants, overall and by gender. Overall, participant age ranged from 18 to 76 (mean 46) years, and 14.9% identified as female, 3.5% non-binary, and 81.6% as male. While 64.5% of participants were White, 13.1% were Black, 8.6% Latinx, 6.8% Asian, 4.0% Native American, and 2.0% Hawaiian/Pacific Islander. The mean social support score was 60.4 (standard deviation [SD] 18.3, range 12–84), and mean HRQoL score was 77.7 (SD 19.2, range 17–100). Female, non-binary, and male participants differed with respect to age, race/ethnicity, and HRQoL, with males being older, more often White, and having higher HRQoL scores. Supplementary Table S1 presents pairwise correlation coefficients for social support, HRQoL, GAD-7, and PHQ-8 scores, which were all significantly correlated (*p* < 0.0001). Supplementary Table S2 presents social support and quality of life domain scores and moderate-to-severe anxiety and depression prevalence, overall and by gender. There were significant differences in reported pain or discomfort, with female participants reporting more pain than non-binary or male participants (mean score 1.8 vs. 1.4 and 1.5, *p* = 0.0015).

**Table 1.** Demographic and Social Characteristics of 397 People Living with HIV

Characteristics	Overall *N* = 397	Female^a^ *N* = 59 (14.9%)	Non-binary^b^ *N* = 14 (3.5%)	Male^c^ *N* = 324 (81.6%)	*p* for comparison
Demographic variables	Mean (SD) or *N* (%)	Mean (SD) or *N* (%)	Mean (SD) or *N* (%)	Mean (SD) or *N* (%)	
Age in years	46.0 (12.0)	44.4 (10.2)	38.7 (10.8)	46.6 (12.2)	0.03
Race/ethnicity					0.002
Asian	27 (6.8%)	2 (3.4%)	1 (7.1%)	24 (7.4%)	
Black	52 (13.1%)	20 (33.9%)	2 (14.3%)	30 (9.3%)	
Hawaiian/Pacific Islander	8 (2.0%)	0	1 (7.1%)	7 (2.2%)	
Latinx	34 (8.6%)	3 (5.1%)	1 (7.1%)	30 (9.3%)	
Native American	16 (4.0%)	2 (3.4%)	0	14 (4.3%)	
White	256 (64.5%)	32 (54.2%)	9 (64.3%)	215 (66.4%)	
Unknown	4 (1.0%)	0	0	4 (1.2%)	
*Social variables*					
Social support score	5.0 (1.5)	5.1 (1.6)	4.9 (1.9)	5.0 (1.5)	0.86
Quality of life score	77.7 (19.2)	71.4 (23.2)	76.3 (15.6)	78.9 (18.4)	0.02
PHQ-8 score	7.8 (5.7)	7.8 (5.8)	10.5 (6.8)	7.6 (5.6)	0.19
GAD-7 score	7.0 (5.9)	7.5 (6.0)	8.7 (7.1)	6.9 (5.8)	0.42
Current living situation more crowded?	47 (11.8%)	11 (18.6%)	2 (14.3%)	34 (10.5%)	0.16

^a^ Includes six transgender women.

^b^ All 14 non-binary individuals had male sex at birth.

^c^ Includes three transgender men.

GAD-7, Generalized Anxiety Disorder-7; PHQ-8, Patient Health Questionnaire-8; SD, standard deviation.

One-way analysis of variance was used to compare continuous variables, and chi-square or Fisher’s exact tests were used to compare categorical variables.

Table 2 presents the results of regression analyses, with the first two columns showing results of models without interaction terms. In bivariable linear regression, social support, gender, depressive symptoms, anxiety symptoms, and crowded living situation were all significantly associated with HRQoL. In multivariable analysis with adjustment for age, gender, depressive symptoms, anxiety symptoms, and crowded living situation, higher social support score remained associated with higher HRQoL score (adjusted *β* = 1.05, 95% confidence interval [95% CI] 0.002, 2.10 per increment in social support score). The multivariable analyses also demonstrated that being male (adjusted *β* = 7.18, 95% CI 2.99, 11.37) was associated with significantly higher HRQoL scores than being female, while being non-binary (adjusted *β* 7.55, 95% CI −1.48, 16.59) was not. Being older (adjusted *β* = −0.17, 95% CI −0.30, −0.04 per year), more depressive symptoms (adjusted *β* = −1.11, 95% CI −1.55, −0.67 per increment in PHQ-8 score), and more anxiety symptoms (adjusted *β* = −1.02, 95% CI −1.45, −0.60 per increment in GAD-7 score) were associated with lower HRQoL scores.

**Table 2.** Linear Regression of Social Support and Quality of Life Among People Living with HIV

	Bivariable		Multivariable		Multivariable with social support by gender interaction	
Characteristics	Beta (95% CI)	*p* Value	Adjusted Beta (95% CI)	*p* Value	Adjusted Beta (95% CI)	*p* Value
Social support	3.64 (2.42, 4.86)	<0.001	1.05 (0.002, 2.10)	0.050	3.84 (1.43, 6.25)	0.002
Age in years	0.05 (−0.11, 0.21)	0.56	−0.17 (−0.30, −0.04)	0.010	−0.18 (−0.31, −0.05)	0.008
Gender identity		0.02		0.004		0.003
Female	Reference		Reference		Reference	
Male	7.54 (2.23, 12.86)		7.18 (2.99, 11.37)		24.53 (10.50, 38.55)	
Non-binary/Genderqueer	4.92 (−6.24, 16.08)		7.55 (−1.48, 16.59)		21.07 (−5.53, 47.67)	
PHQ-8 score	−2.01 (−2.28, −1.75)	<0.001	−1.11 (−1.55, −0.67)	<0.001	−1.07 (−1.51, −0.63)	<0.001
GAD-7 score	−1.92 (−2.18, −1.66)	<0.001	−1.02 (−1.45, −0.60)	<0.001	−1.06 (−1.49, −0.64)	<0.001
Current living situation more crowded?	−8.48 (−14.36, −2.60)	0.005	−2.21 (−6.97, 2.55)	0.36	−1.68 (−6.45, 3.09)	0.49
Interaction term (social support and gender)						0.040
Female					Reference	
Male					−0.28 (−0.50, −0.06)	
Non-binary/Genderqueer					−0.22 (−0.64, 0.20)	

CI, confidence interval.

**FIG. 1.**
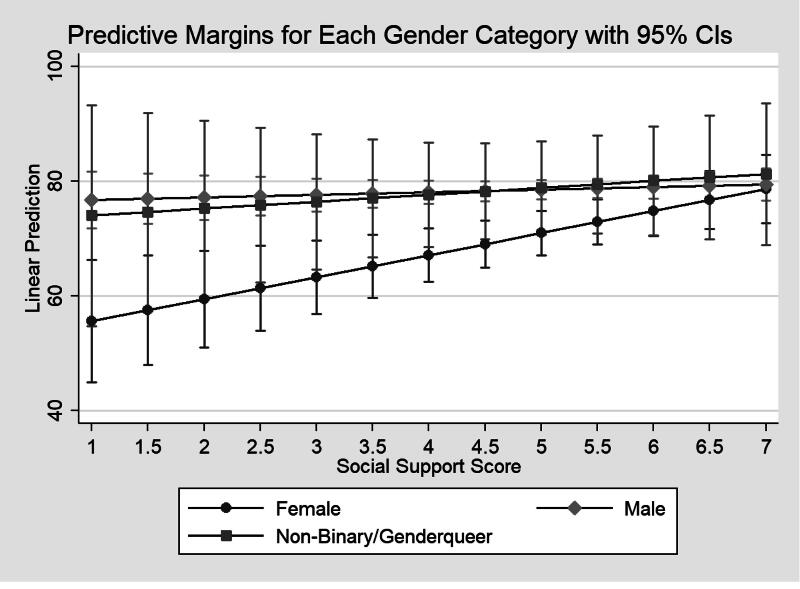
Predicted quality of life scores at different levels of social support for each gender category. Stata’s “marginsplot” command was used to graph social support on the *x*-axis and predicted quality of life scores with 95% confidence intervals (CIs) on the *y*-axis. Predicted margins for females are indicated with a circle, for males with a diamond, and for non-binary/genderqueer individuals with a square.

When interaction terms were added for modeling, age did not moderate the association between social support and HRQoL in the model with only the social-support-by-age interaction (*p* = 0.114) or in a model with both interaction terms (*p* = 0.148, not presented). In the multivariable model without the social-support-by-age interaction term, adjusting for confounding variables, gender moderated the association between social support and HRQoL (see column 3, Table 2). The association between social support and HRQoL (adjusted *β* = −0.28, 95% CI: −0.50, −0.06) was significantly moderated by gender, with a weaker association observed among men compared with women. Non-binary/genderqueer individuals had HRQoL and slopes for the relationship between social support and HRQoL that did not differ significantly from those of females, with estimates closer to those of males than females. Figure 1 illustrates the marginal plot of the relationship between social support and HRQoL, stratified by gender. The plot demonstrates that while HRQoL increases with higher levels of social support across all groups, the slope is considerably steeper for women compared with male and non-binary participants. Predicted HRQoL score at the lowest possible social support score (1) was 55.6 for females, 76.7 for males, and 74.0 for non-binary people. At the highest possible social support score (12), the predicted HRQoL score for females (78.6), was very similar to predicted scores for males and non-binary individuals (79.4 and 81.2, respectively). Higher social support remained associated with higher HRQoL score (adjusted *β* = 3.84, 95% CI 1.43, 6.25 per increment in social support score). In addition, being older (adjusted *β* = −0.18, 95% CI −0.31, −0.05 per year), more depressive symptoms (adjusted *β* = −1.07, 95% CI −1.51, −0.63 per increment in PHQ-8 score), and more anxiety symptoms (adjusted *β* = −1.06, 95% CI −1.49, −0.64 per increment in GAD-7 score) were associated with lower HRQoL scores.

In a sensitivity analysis in which all individuals were classified as having either male or female sex at birth, results were very similar other than the omission of the non-binary/genderqueer group (Supplementary Table S1). The adjusted *r*-squared was almost identical (0.4176 for the model with gender and 0.4189 for the model with sex at birth), and the interaction between sex at birth and social support was significant (adjusted *β* −0.29, 95% CI −0.52, −0.06 for males compared with females).

## Discussion

This study revealed a positive association between social support and HRQoL among PWH during the COVID pandemic, with higher levels of perceived social support associated with higher self-rated HRQoL. This relationship persisted after adjustment for depressive symptoms and anxiety, which themselves were strong negative predictors of self-rated HRQoL. Importantly, gender moderates the relationship between social support and HRQoL. Specifically, at low levels of social support, female participants reported lower HRQoL compared with male and non-binary individuals, suggesting that women may be more vulnerable to reductions in social support. While age was an independent predictor of lower HRQoL, it did not moderate the association between social support and HRQoL.

Several studies of PWH have shown that social support directly relates to quality of life for individuals, in line with this study’s finding that HRQoL increased by 0.32 points for every 1-point increase in social support score in our final adjusted model. For example, a study of 120 PWH from Toronto, Canada reported a positive association between emotional social support and quality of life assessed by the Fanning Quality of Life Scale, a health-related measure.^6^ Also consistent with our current findings, in a qualitative analysis of in-depth interviews with a subsample of participants from the current study, individuals reported feelings of loneliness or lack of social support that resulted in a negative impact on quality of life.^11^ While social support is generally important for maintaining HRQoL, its importance may increase during stressful events such as the COVID-19 pandemic.

While we found that social support was an independent predictor of HRQoL, our results also demonstrate that mental health (*i.e.,* PHQ-8 and GAD-7 scores) was a strong predictor of HRQoL for our study participants. Abundant literature from before the COVID pandemic supports associations between mental health and quality of life. For example, a prepandemic study of 1241 individuals seeking care in 28 primary care centers in Spain found a strong relationship between depressive symptoms and lower quality of life, assessed using the World Health Organization Quality-of-Life Short Form (WHO QOL-BREF).^21^ A separate study of 2864 adults receiving care for HIV in the United States found that PWH with a probable mood disorder diagnosis had significantly lower quality of life scores (assessed using 28 items covering physical, mental, and social domains of life) than those without the same symptoms.^8^ The pandemic likely exacerbated this relationship. An editorial written early in the pandemic (April 2020) warned of the mental health consequences of COVID-19 and the measures required to prevent its spread, predicting a drastic increase in anxiety, depression, and loneliness.^22^ In our published analysis of data from the current study among PWH in Western Washington, we found that COVID-related stress remained positively associated with depression and anxiety symptoms during the pandemic, even after adjustment for prepandemic symptoms^10^; our HRQoL measure was not included in that study.

While some studies have found that age impacts the relationship between social support and quality of life, we did not find any moderation by age. However, higher age did predict a lower HRQoL. In a study of older adults living in England, Zaninotto et al. found that quality of life was poorer for older than younger respondents and associated with both decreased number of friends and low positive social support.^23^ In contrast, we found that gender was both an important predictor of HRQoL and a moderator of the relationship between social support and HRQoL. Specifically, we found that women were more sensitive to the relationship between social support and HRQoL than men or nonbinary individuals. There are several reasons why this might be so. In a study of young adults across Norway, Johansen et al. found an association between low social support and mental distress that was significant for young women only. They hypothesized that this difference may be related to gender-specific stressors that develop from the socially defined roles of women and men.^24^ Of interest, a literature review on gender differences in social support and physical health found that women report receiving more emotional support than men and are more likely than men to be both support providers and recipients.^25^

Our findings are also consistent with prior studies that found that women are more likely to report lower quality of life than men, both generally and among PWH. For example, in a study conducted among the general population in England, PWH had lower HRQoL compared with the general population, and being female was associated with lower HRQoL than being male.^26^ A systematic review of 12 studies regarding the impact of COVID-19 on the HRQoL of patients also found that women had lower HRQoL scores than men.^27^ Of note, one of the ways our findings add to the body of work on gender and quality of life is by the inclusion of non-binary participants, who are frequently left out of research studies. All 14 non-binary participants in this study were assigned male sex at birth, which aligns with the fact that their results tracked closer to those of males than of females in this study.

The findings of this study have implications for interventions to mitigate the effects of future stressful events. If a new pandemic or any other significantly stressful life event were to occur, our results suggest that social support should be prioritized as an approach to buffer negative impacts on quality of life. A study of 69,066 participants who responded to the COVID-19 Participant Experience (COPE) Survey in 2022 found that social support, including emotional/informational support, positive social interactions, and tangible support, was associated with significantly reduced odds of depression,^28^ which can be a cause for reduced quality of life. Similar to our study, women were more impacted than men or non-binary individuals, and the effects of gender (and age, in the COPE study) were attenuated at higher levels of social support.^28^

Our study has several strengths. First, we were able to enroll a diverse sample of 397 PWH for online participation during a period when in-person research was not permitted due to COVID-19 social distancing. Second, the sample size was adequate to evaluate interaction terms for the continuous predictor and outcome we used. Third, responses were collected online using REDCap and not in a face-to-face interview, reducing the chance of self-presentation bias in responses. Our study also has its limitations. First, the generalizability of the results is limited as the sample was restricted to individuals who were enrolled in care at a participating clinic, were included in the UW HIV patient registry, and were willing to participate in the online survey. Participants also had to have internet access, which led to the exclusion of some otherwise eligible individuals without these resources. Second, while the proportion of women in our sample was similar to that in the UW HIV patient registry in 2020 (14.9% vs. 14.3%, respectively [unpublished data]), the number of women in our study was low. Third, some variables that may have been helpful in hindsight, such as household composition and access to online social support, were not collected. Fourth, there was the potential for recall bias in the self-reported responses from participants. Finally, our study design was cross-sectional, and we are not able to infer causal relationships based on these data.

## Conclusion

In conclusion, we found a positive association between social support and HRQoL among PWH in Western Washington State during the COVID pandemic. This relationship was independent of depressive symptoms and anxiety and was moderated by gender. While older age predicted lower HRQoL, age did not moderate the relationship between social support and HRQoL. Individuals, especially females, and organizations providing services to PWH should prioritize social support as an important means to promote quality of life, especially during public health emergencies.

## Abbreviations Used

CDC: Centers for Disease Control and Prevention
CI: confidence interval
GAD-7: Generalized Anxiety Disorder-7
HIV: human immunodeficiency virus
HRQoL: health-related quality of life
PHQ-9: Patient Health Questionnaire-9
PWH: people living with HIV
REDCap: Research Electronic DataCapture
SD: standard deviation
UW: University of Washington
