# Supplementary material for: Gender Differences in the Relationship Between Social Support and Quality of Life Among People Living with HIV During the COVID-19 Pandemic
Source: Womens Health Rep (New Rochelle). 2024 Nov 25;5(1):916–24. doi: 10.1089/whr.2024.0112 (PMC12722283; doi:10.1089/whr.2024.0112)
Supplement: Supplementary Table S1 [file whr.2024.0112_supp_tables1.docx]

**Supplemental Table 1. Linear Regression of Social Support and Quality of Life among People Living with HIV: Sensitivity Analysis using Sex at Birth**

|  | Bivariable | | Multivariable | | Multivariable with Social Support by Gender Interaction | |
| --- | --- | --- | --- | --- | --- | --- |
| Characteristics | Beta (95% CI) | P-Value | Adjusted Beta (95% CI) | P-Value | Adjusted Beta (95% CI) | P-Value |
| Social support | 3.64 (2.42, 4.86) | <0.001 | 1.12 (0.06, 2.17) | 0.04 | 4.13 (1.56, 6.70) | 0.002 |
| Age in years | 0.05 (-0.11, 0.21) | 0.56 | -0.17 (-0.30, -0.04) | 0.01 | -0.17 (-0.30, -0.04) | 0.01 |
| Gender identity |  |  |  |  |  |  |
| Female | Reference |  | Reference |  | Reference |  |
| Male | 7.04 (1.62, 12.46) | 0.01 | 7.04 (2.77, 11.32) | 0.001 | 25.41 (10.47, 40.36) | 0.001 |
| PHQ-8 score | -2.01 (-2.28, -1.75) | <0.001 | -1.12 (-1.56, -0.68) | <0.001 | -1.09 (-1.52, -0.65) | <0.001 |
| GAD-7 score | -1.92 (-2.18, -1.66) | <0.001 | -1.01 (-1.43, -0.58) | <0.001 | -1.04 (-1.46, -0.61) | <0.001 |
| Current living situation more crowded | -8.48 (-14.36, -2.60) | 0.005 | -2.32 (-7.07, 2.44) | 0.34 | -1.88 (-6.62, 2.85) | 0.43 |
| Interaction term (social support and gender) |  |  |  |  |  | 0.012 |
| Female |  |  |  |  | Reference |  |
| Male |  |  |  |  | -0.29 (-0.52, -0.06) |  |

CI = Confidence Interval, GAD-7 = Generalized Anxiety Disorder-7, PHQ-8 = Patient Health Questionnaire-8
