# Supplementary material for: Gender Differences in the Relationship Between Social Support and Quality of Life Among People Living with HIV During the COVID-19 Pandemic
Source: Womens Health Rep (New Rochelle). 2024 Nov 25;5(1):916–24. doi: 10.1089/whr.2024.0112 (PMC12722283; doi:10.1089/whr.2024.0112)
Supplement: Supplementary Table S2 [file whr.2024.0112_supp_tables2.docx]

**Supplemental Table 2. Social Support, Quality of Life, and Mental Health Symptoms among 397 People Living with HIV**

| Demographic variables | Overall  N = 397 | Female^a^  N = 59 (14.9%) | Non-binary^b^  N = 14 (3.5%) | Male^c^  N = 324 (81.6%) | P for comparison |
| --- | --- | --- | --- | --- | --- |
|  | Mean (SD) or N (%) | Mean (SD) or N (%) | Mean (SD) or N (%) | Mean (SD) or N (%) |  |
| Social support domain scores |  |  |  |  |  |
| Significant other | 5.3 (1.8) | 5.5 (1.9) | 5.4 (2.1) | 5.3 (1.8) | 0.77 |
| Family | 4.7 (1.9) | 5.0 (1.9) | 4.4 (2.2) | 4.7 (1.9) | 0.45 |
| Friends | 5.1 (1.6) | 4.9 (1.8) | 4.9 (2.0) | 5.1 (1.6) | 0.55 |
| Quality of life domain scores |  |  |  |  |  |
| Mobility | 1.2 (0.4) | 1.3 (0.5) | 1.1 (0.4) | 1.2 (0.4) | 0.18 |
| Self care | 1.1 (0.3) | 1.2 (0.4) | 1.1 (0.3) | 1.1 (0.3) | 0.14 |
| Usual activities | 1.3 (0.5) | 1.4 (0.5) | 1.4 (0.5) | 1.3 (0.5) | 0.28 |
| Pain or discomfort | 1.5 (0.6) | 1.8 (0.7) | 1.4 (0.5) | 1.5 (0.6) | 0.0015 |
| Anxiety or depression | 1.8 (0.7) | 1.9 (0.7) | 2.1 (0.5) | 1.8 (0.7) | 0.08 |
| Moderate-to-severe depression (PHQ-8 ≥10) | 138 (34.8) | 22 (37.3) | 7 (50.0) | 109 (33.6) | 0.41 |
| Moderate-to-severe anxiety (GAD-7 ≥10) | 114 (28.7) | 21 (35.6) | 4 (28.6) | 89 (27.5) | 0.44 |

GAD-7 = Generalized Anxiety Disorder-7, PHQ-8 = Patient Health Questionnaire-8, SD = Standard Deviation

Note: One-way analysis of variance was used to compare continuous variables and Chi square or Fisher exact tests were used to compare categorical variables.

^a^ Includes 6 transgender women

^b^ All 14 non-binary individuals had male sex at birth

^c^ Includes 3 transgender men
