# Supplementary material for: Gender Differences in the Relationship Between Social Support and Quality of Life Among People Living with HIV During the COVID-19 Pandemic
Source: Womens Health Rep (New Rochelle). 2024 Nov 25;5(1):916–24. doi: 10.1089/whr.2024.0112 (PMC12722283; doi:10.1089/whr.2024.0112)
Supplement: Supplementary Table S3 [file whr.2024.0112_supp_tables3.docx]

**Supplemental Table 3. Pairwise Correlations between Social Support, Quality of Life, and Mental Health Scores**

|  | Social support | Quality of Life | GAD-7 score |
| --- | --- | --- | --- |
| Quality of life | 0.2875, p <0.0001 |  |  |
| GAD-7 score | -0.2755, p <0.0001 | -0.5852, p <0.0001 |  |
| PHQ-8 score | -0.3377, p <0.0001 | -0.5995, p <0.0001 | 0.7957, p <0.0001 |

GAD-7 = Generalized Anxiety Disorder-7, PHQ-8 = Patient Health Questionnaire-8
